# A Systematic Review of Organic Versus Conventional Food Consumption: Is There a Measurable Benefit on Human Health?

**DOI:** 10.3390/nu12010007

**Published:** 2019-12-18

**Authors:** Vanessa Vigar, Stephen Myers, Christopher Oliver, Jacinta Arellano, Shelley Robinson, Carlo Leifert

**Affiliations:** 1 NatMed Research, Southern Cross University, Lismore NSW 2480, Australia; vanessa.vigar@scu.edu.au (V.V.); christopher.oliver@scu.edu.au (C.O.); shelley.robinson@scu.edu.au (S.R.); 2 Integria Healthcare, Eight Mile Plains QLD 4113, Australia; 3 School of Health and Human Sciences, Southern Cross University, Lismore NSW 2480, Australia; jacinta.arellano@scu.edu.au; 4 Centre for Organics Research, Southern Cross University, Lismore NSW 2480, Australia; 5 Oliver Nutrition Pty Ltd, Lismore NSW 2480, Australia

**Keywords:** health outcomes, organic, organic diet, pesticide-free, sustainable diet

## Abstract

The current review aims to systematically assess the evidence related to human health outcomes when an organic diet is consumed in comparison to its conventional counterpart. Relevant databases were searched for articles published to January 2019. Clinical trials and observational research studies were included where they provided comparative results on direct or indirect health outcomes. Thirty-five papers met the criteria for inclusion in the review. Few clinical trials assessed direct improvements in health outcomes associated with organic food consumption; most assessed either differences in pesticide exposure or other indirect measures. Significant positive outcomes were seen in longitudinal studies where increased organic intake was associated with reduced incidence of infertility, birth defects, allergic sensitisation, otitis media, pre-eclampsia, metabolic syndrome, high BMI, and non-Hodgkin lymphoma. The current evidence base does not allow a definitive statement on the health benefits of organic dietary intake. However, a growing number of important findings are being reported from observational research linking demonstrable health benefits with organic food consumption. Future clinical research should focus on using long-term whole-diet substitution with certified organic interventions as this approach is more likely to determine whether or not true measurable health benefits exist.

## 1. Introduction 

The global marketplace of organics has grown rapidly over the last few decades and consumer demand for organic products is increasing globally, with approximately 80 billion Euros ($92 billion USD) spent on organic products annually [1]. A recent report from the Research Institute of Organic Agriculture (FiBL) and IFOAM Organics International, shows a 14.7% increase in organic farmland from 2014 to 2015, totalling 50.9 million hectares, with Australia having the largest amount of agricultural land at 22.7 million hectares [2]. Organic food items most often consumed in Europe are organic baby foods followed by organic eggs, fruit and vegetables, then dairy products, with organic dairy reaching market shares of around 10 percent of overall sales in some European countries [2]. In the United States, fruit and vegetables make up the largest areas of organic food sales, followed by dairy products [3]. The reasons consumers are increasingly choosing organic over conventional food products are varied, including many reasons beside personal health and wellbeing, such as environmental concerns or animal welfare impact. However, the major determinants behind consumer purchase of organic products, is the belief that organic food is healthier or has a superior nutritional profile [4,5,6].

Regular consumers of organic food are most likely to be female, health-conscious, physically active, and in the higher brackets of education and income than their non-organic consuming counterparts [7,8]. They are also more likely to have a higher ratio of plant to animal foods, with a strong relationship between vegetarian/vegan consumers and organic consumption [7,9]. This consumer group generally has an increased wholefood dietary intake, as a result of both the general ethos of organic consumers (i.e., preference over processed/ultra-processed foods), and restricted use of additives in organic processed foods. Diet composition between organic and non-organic consumers may, therefore, be quite different. 

The notion that organic food may be healthier has some support. Although there appears to be little variation between organic and conventional food products in terms of macro nutritional value (protein, fat, carbohydrate and dietary fibre), other compositional differences have been demonstrated. These include higher antioxidant concentrations (particularly polyphenols) in organic crops [10]; increased levels of omega-3 fatty acids in organic dairy products [11,12,13]; and improved fatty acid profiles in organic meat products [14,15]. These compositional differences are comprehensively discussed in several recent reviews [16,17,18,19]. There is preliminary evidence to suggest that these compositional differences may have an effect on plasma levels of certain nutrients including magnesium, fat-soluble micronutrients (α-carotene, *β*-carotene, lutein, and zeaxanthin), and fatty acids (linoleic, palmitoleic, γ-linolenic, and docosapentaenoic acids) [20]. Any possible clinical effects of such differences need further investigation.

Likely to be of more importance than compositional differences between the two, is what organic foods *do not* contain. Organic foods have been shown to have lower levels of toxic metabolites, including heavy metals such as cadmium, and synthetic fertilizer and pesticide residues [10,17]. Consumption of organic foods may also reduce exposure to antibiotic-resistant bacteria [19].

The long-term safety of pesticide consumption through conventional food production has been questioned, with evidence from long-term cohort studies covering areas ranging from possible neurotoxicity to endocrine disruption [21]. A number of widely used pesticides have been banned retrospectively only when unexpected negative health impacts have been identified [22,23]. From a regulatory perspective, dietary intake of pesticides is not considered to pose a health risk to consumers as long as individual pesticide concentrations in foods are below the Maximum Residue Level (MRL). Surveys conducted by both the European Food Safety Authority and the United States Department of Agriculture show that the vast majority of foods contained individual pesticide levels below the MRL, at 1.7% and 0.59%, respectively, found to exceed the limits. It was also found that 30.1% and 27.5%, respectively, of food samples analysed contained multiple pesticide residues [24,25]. One of the main criticisms of current regulatory pesticide approval processes is that they do not require safety testing of pesticide mixtures or formulations of pesticides [23,26,27]. There is considerable controversy about health risks posed by chronic low-level dietary pesticide exposure [28,29,30], and whilst lower levels of pesticide residue excretion is consistently observed during organic diet intakes [31,32,33,34], there is uncertainty around how this may impact the health of the consumer. 

The last systematic review into the effect of organic food consumption on health was conducted by Dangour et al. in 2010 [35], which was limited to strict inclusion criteria of organic interventions, and Smith-Spangler et al. in 2012 [19], which contained only minimal focus on the human health effects of organic food, and a broader focus on nutritional content of organically and conventionally grown food and food safety. Although there have been other more recent reviews on the effects of organic diet on broader aspects of health [16,17,18,21], none have been systematic. The literature has expanded since these earlier systematic reviews, with many cohort and cross-sectional studies being published which compare organic versus conventional dietary intake on a range of health outcomes. Dangour et al. (2010) included 12 reports overall, of which eight were human studies (six clinical trials, one cohort study, 1 cross-section study), and four that reported animal or in vitro research. The Smith-Spangler et al. (2012) report was more comprehensive, including 17 human studies (in addition to 223 studies of comparative nutrient/contaminant profiles).

The present systematic review was designed to assess the breadth of evidence related to human health outcomes when an organic diet is consumed in comparison to its conventional counterpart. This review reports results from 35 studies including both clinical trial and observational research and includes substantially more papers than previous systematic reviews on this topic. This review does not include a comparison of nutritional quality between production types, safety of organic food, or human studies where environmental pesticide exposure is the focus.

## 2. Methods

### 2.1. Literature Search 

This systematic review has been conducted in accordance with the guidelines of the Preferred Reporting Items of Systematic Reviews and Meta-analysis (PRISMA) statement [36]. 

Relevant studies were identified by a systematic search from the Cochrane, MEDLINE, EMBASE, and TOXNET databases for articles published in January 2019. Relevant keywords included terms related to organic dietary intake in combination with words relevant to health outcomes (i.e., asthma, eczema, obesity, diabetes). Search terms were amended slightly for each database. Articles with English titles and abstracts were considered for inclusion. The search strategy was developed by two authors (SM and VV) and was performed by VV in January 2019. Additional publications were identified from the reference lists of obtained articles that were included in the review. Refer to Supplementary Figure S1 (see online Supplementary Material).

All articles that compared organic versus conventional dietary intake in relation to a direct or an indirect health outcome were included. We did not set out to limit paper inclusion by including a strict definition of organic intake, but accepted all papers that self-identified as representing comparative information on health outcomes from organic versus conventional diets. In doing so, we set out a priori to ensure we obtained a comprehensive snapshot of the available literature in this area. 

### 2.2. Study Eligibility Criteria

#### 2.2.1. Population

Only human feeding studies were included. Studies including infant participants measured from the second trimester of pregnancy were included where the mother gave dietary information during pregnancy. 

#### 2.2.2. Intervention

Any clinical trial where organic food items were taken to replace non-organic food items, or observational studies where there was a comparison between organic and non-organic dietary intake were included. This encompassed individual food or drink replacement, through to entire diet substitution. Observational research was accepted where dietary intake was classified according to level of organic food within individual dietary groups or whole diet. 

#### 2.2.3. Outcome

Clinical trials were included where they provided comparative results on direct or indirect health outcomes. Cohort studies were included where associations with development of disorder or disease were reported, or if they provided comparisons of biological samples across organic versus conventional dietary intake groups. 

#### 2.2.4. Study Designs

Types of studies included were randomised controlled trials (RCT), non-controlled trials, prospective or retrospective cohort studies, case-control studies and cross-sectional studies. 

#### 2.2.5. Exclusion Criteria

Articles were excluded if they were not specifically examining the effect of organic dietary intake with conventional dietary intake, or if they did not report on human biomarkers related to health, or disease development. Articles were excluded if they were concerned with occupational exposure to agricultural chemicals or domestic use of pesticides and unrelated to dietary consumption of organic versus non-organic foods.

### 2.3. Data Extraction

Two reviewers independently reviewed full articles for inclusion based on relevance to the study question and eligibility criteria. One reviewer (VV) extracted data from included studies, which was checked by a separate reviewer (SM). The details are presented in Table 1 and Table 2, using the following parameters: (i) author and year of publication; (ii) study population including country of origin and key demographic detail; (iii) sample size; (iv) study design and duration of intervention/exposure; (vi) exposure to organic diet and comparator; (vii) outcomes assessed; (viii) results; (ix) organic definition.

### 2.4. Assessment of Risk of Bias

The Cochrane Risk of Bias Assessment Tool was used to assess likelihood of bias in each clinical trial publication [67]. The Newcastle–Ottawa Quality Assessment Form for Cohort Studies was used to assess the likelihood of bias in cohort studies, and the Specialist Unit for Review Evidence (SURE) checklist was used for the critical appraisal of cross-sectional studies [68,69]. All assessments were conducted by at least two authors, with differences settled by discussion. Summary tables detailing results of bias assessments are presented in Supplementary Figure S2. 

## 3. Results

### 3.1. Study Selection and Characteristics

**Figure 1:**
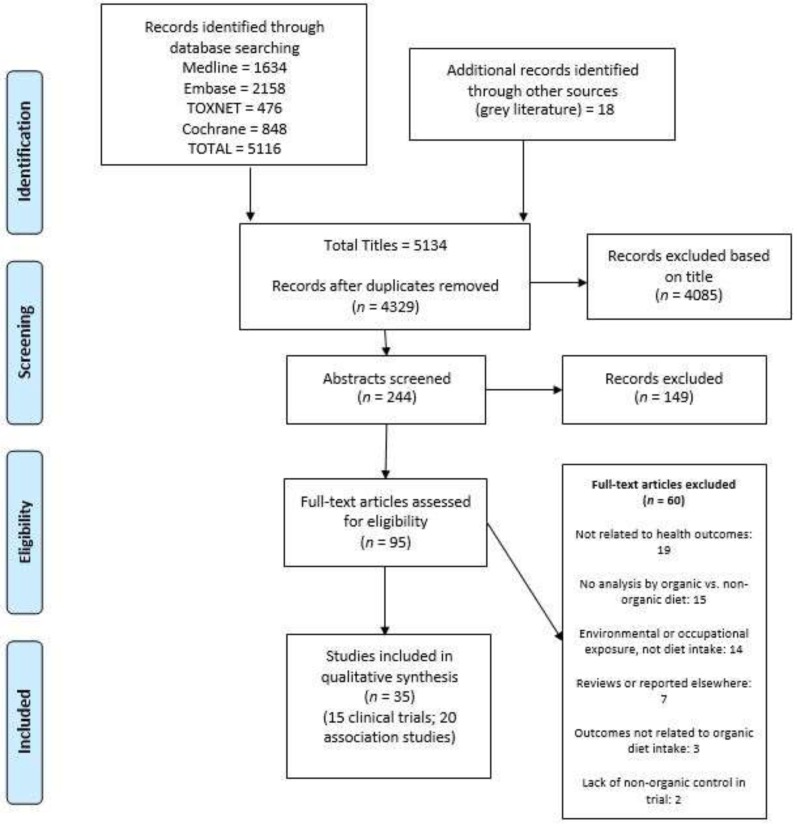
PRISMA flow diagram of study selection [36].

The Preferred Reporting Items for Systematic Reviews and Meta-Analyses (PRISMA) flow diagram detailing the article selection process is shown in Figure 1. Searches identified 4329 potentially relevant articles, of which 4234 were excluded after initial screening of title and/or abstract. The remaining 95 full-text publications were assessed, of which a further 60 publications were excluded. 

Thirty-five papers met the criteria for inclusion in this review. Of these, 15 publications reported on 13 clinical trials—three of which were parallel-arm randomised controlled trials (RCT), with the remaining studies utilising a crossover design. In observational studies, 20 publications reported on 13 cohorts. The studies were all published in English. The majority of the clinical trials were conducted in Europe—Germany (2), Denmark (2), Italy (2), France (1), and Switzerland (1), with other countries including: the United States (2), Turkey (1), Brazil (1), and Australia (1). Observational research studies were on cohorts from the United States, United Kingdom, Norway, France, Denmark, Netherlands, and Sweden.

### 3.2. Clinical Trials (Single Food/Drink Item Substitution)

Several studies investigated the effect of replacing a single non-organic food or drink item with its organic counterpart. Three of the trials utilised an acute dose setting (red wine, apples or grape juice) in a crossover design [40,42,48], while others were based on the daily consumption of the food item (tomatoes and derived purees, carrots or apples) for a period of 2–4 weeks [37,38,39]. Those studies looking at nutrient levels (i.e., carotenoids, polyphenols) [37,38,39] in biological samples (blood or urine), did not find any significant differences in the levels of these markers as a result of the organic intervention. 

Other single-item substitution studies measured antioxidant capacity, or DNA damage in biological samples [38,39,40,42,48]. There were no significant between-group differences in these biomarkers in any of the studies.

### 3.3. Clinical Trials (Whole Diet Substitution)

Eight crossover trials (reported in nine publications) investigated the effect of whole diet replacement from conventional to organic (or at least >80% in one study) for a time period ranging from 4 or 5 days in children [31,43,44] to up to 22 days in adult populations [34,41,45,46,47,49]. 

Four of these trials (two in children and two in adults) measured changes in pesticide excretion through urine [31,34,43,44,49]. All of these trials demonstrated a significant difference in the amount of pesticide metabolites excreted during the different phases of the diet interventions. The reduction was, in most cases, dramatic (up to 90% reduction during organic phase) and occurred within a short time frame of only a few days. 

The remaining trials were all conducted in adult populations and measured antioxidant capacity and flavonoid excretion [41]; carotenoids [47]; or antioxidant capacity, changes to body composition, lipids and inflammatory markers [45,46].

Similar to the results from clinical trials replacing single food items, individual flavonoid and carotenoid excretion appeared to reflect the content of the foods consumed (i.e., a higher quercetin, carotenoid and kaempferol level was shown in organic produce in comparison to conventional produce given as part of the diets, and this was reflected in the urinary output) [41,47]. 

Two studies completed by the same research group in Italy looked at the effects of a Mediterranean diet intervention (non-organic phase followed by organic phase). An initial pilot study of 10 people [45] and a following larger cohort study of 150 people (100 healthy and 50 with chronic kidney disease (CKD)) [46] provided a two-stage intervention, with a controlled Mediterranean diet (MD) for 14 days followed by the same diet for a further 14 days using organic rather than conventional foodstuffs.

The pilot study found an increased antioxidant effect (from 2.25 to 2.75 mM trolox equivalents) after 14 days MD and after 14 days organic MD, respectively, with no baseline measure provided. The authors also showed a generally higher antioxidant level in the organic foods eaten in comparison to non-organic. In the larger study, in both healthy and CKD patients there was a highly significant effect on body weight reduction and improved body composition seen through dual-energy X-ray absorptiometry (DXA) and bio-impedance analysis (BIA) between the two time points (end of conventional MD and end of organic MD). Inflammatory markers (hs-CRP, IL-1, IL-6, IFN-γ and homocysteine) all showed a statistically significant decrease between the same time-points for the healthy group, whilst only hs-CRP and homocysteine were significantly decreased in the CKD group.

### 3.4. Observational cohort studies

From a total of 20 publications including 13 cohorts, seven prospective cohorts were identified, with the majority involving mother/child pairs. These included the Norwegian Mother and Child Cohort Study [55,56]; KOALA Birth Cohort [58,59,60]; ALLADIN study [61]; PELAIGE Mother–Child Cohort [62] and the EARTH study [52]. Two adult-only cohorts involved development of cancer incidence in the Million Women Study [65], and self-reported health factors in the Nutri-Net Santé Cohort Study [20,53,63,64]. A retrospective case-control study in a mother–child cohort was also included [57].

Several of the identified studies provided cross-section data only. These include comparisons of organic and conventional diets on sperm quality/content [50,51]; breast milk composition [66]; and urinary pesticide excretion [32,33].

For ease of reporting, all of the observational studies have been separated into subject areas. Firstly, looking at potential influence on foetal development (effect on sperm, fertility, and birth defects, pre-eclampsia); breast milk studies; development of allergies in children; urinary pesticide excretion; cancer development incidence; and changes in nutritional biomarkers in adults. 

#### 3.4.1. Sperm and Fertility

Two investigations examined the association between sperm health in Danish organic farmers. The first compares the organic farmers to non-organic farmers and shows a significantly lower proportion of morphologically normal spermatozoa in the non-organic group, but no significant difference in relation to 14 other semen parameters [51]. The other compares the organic farmers to a control group of airline pilots, finding a higher sperm concentration among organic farmers (increased by 43.1%, 95% CI 3.2 to 98.8%), with no differences seen in seminal volume, total sperm count, and sperm morphology [50]. 

The Environment and Reproductive Health (EARTH) study examined associations between high or low dietary pesticide exposure in a group of women using assisted reproduction technology (ART) at the Massachusetts General Hospital Fertility Center [52]. They compared pregnancy/birth outcomes from 325 women (contributing 541 ART cycles) against a dietary pesticide score. They found high-pesticide residue fruit and vegetable (FV) intake was inversely associated with probability of clinical pregnancy and live birth per initiated cycle. Compared with women in the lowest quartile of high-pesticide residue FV intake (<1 serving/day), women in the highest quartile (≥2.3 servings/day) had 18% (95%CI 5%–30%) lower probability of clinical pregnancy and 26% (95%CI 13%–37%) lower probability of live birth. High-pesticide residue FV intake was positively associated with probability of total pregnancy loss. 

#### 3.4.2. Mother–Child cohorts 

The Norwegian Mother and Child Cohort Study (MoBa) investigated associations between an organic diet and conventional diet during pregnancy and the development of pregnancy complications, including pre-eclampsia [56] and incidence of the rare reproductive abnormalities in infant boys—hypospadias or cryptorchidism [55]. Women who reported to have eaten organic vegetables ‘often’ or ‘mostly’ (*n* = 2493, 8.8% of study-sample) were found to have a lower risk of pre-eclampsia than those who reported ‘never/rarely’ or ‘sometimes’ (OR = 0.76, 95%CI 0.61, 0.96). A lower prevalence of hypospadias with any organic consumption, in particular organic vegetables, was found, with no difference for cryptorchidism. This prospective study included 35,107 mothers of male infants in Norway, with organic food in six food groups assessed by food frequency questionnaires (FFQ) [55]. Whole diet composition was considered using slightly different methods in each of these analyses; therefore, residual confounding may exist between the results reported. In a smaller case-control study, retrospective data were collected from mothers of 306 infant males who were operated on for hypospadias matched to 306 mothers of healthy infant males in Denmark. No difference was found for total organic consumption, but increased odds for hypospadias were found specifically when non-organic milk/dairy consumption was combined with frequent consumption of high-fat dairy products (adjusted OR = 2.18, 95%CI 1.09, 4.36) [57].

The PELAIGE study in France (*n* = 1505) was a prospective cohort study that examined the incidence of otitis media during early childhood, finding frequent intake of organic diet during pregnancy was associated with decreased risk of having at least one episode of otitis media (OR = 0.69, 95%CI 0.47, 1.00) [62]. A sub-group analysis measuring pesticide residues in urine, found the presence of dealkylated triazine metabolites was positively associated with recurrent otitis media (OR = 2.12, 95%CI 1.01, 4.47). 

The influence of organic food consumption as part of an anthroposophical lifestyle in pregnancy and early childhood has been discussed following two major studies—the KOALA birth cohort in the Netherlands [60,70,71], and the ALADDIN birth cohort in Sweden [61]. In the KOALA cohort (*n* = 2764), consumption of organic dairy products was associated with lower eczema risk (OR = 0.64, 95%CI 0.44, 0.93), but there was no association for other food types or overall organic content of diet with the development of eczema, wheeze or atopic sensitisation. No statistically significant associations were observed between organic food consumption and recurrent wheeze (OR = 0.51, 95%CI 0.26, 0.99) during the first 2 years of life [60]. In the ALADDIN study (*n* = 330), a markedly decreased risk of sensitisation during the first 2 years of life was seen in children of anthroposophic families compared with children of non-anthroposophic families with adjusted OR of 0.25 (95%CI 0.10, 0.64, *p* = 0.004) [61].

It is important to note that organic food consumption is only one of several food-specific differences that are a key part of the anthroposophic lifestyle (see discussion).

#### 3.4.3. Early Childhood 

Minimal changes were seen in breastmilk composition in the KOALA birth cohort study, with increased rumenic acid and a trend for increased trans-vaccenic acid in quartiles of highest organic consumption [58]. No difference was seen in trans fatty acid content within the same cohort [60]. An American study examining milk and urine samples of lactating women for glyphosate and aminomethylphosphonic acid (AMPA) did not find any evidence of these chemicals in the breast milk of conventional or organic food consumers [66].

Similar to the findings in urinary output of pesticides found in clinical trial research, cross-sectional analysis of organophosphorus metabolites in children (*n* = 39) show that those consuming organic foods have considerably lower levels of dimethyl metabolites in their urine than those consuming conventional diets (0.03 and 0.17 μmol/L, *p* < 0.001), respectively [33]. 

#### 3.4.4. Adult Research

The Nutri-Net Santé Cohort has analysed data from 62,224 participants enrolled in France, through an internet-based survey, with information on frequency of organic food consumption and repeated anthropometric data. The data was predominantly self-reported. An increase in the organic score was associated with a lower risk of being overweight (OR = 0.77, 95%CI 0.68, 0.86, *p* < 0.0001). The association remained strong and highly significant, with a reduction in the risk of obesity of 37% after a 3.1-year follow-up [63]. A cross-section of the cohort (*n* = 8174) examined for metabolic syndrome also detailed positive impact of an organic diet with an adjusted prevalence ratio of 0.69 (95%CI 0.61, 0.78) when comparing the third tertile of organic food in the diet with the first one (*p* < 0.0001) [64]. Additionally, a nested case-control study (*n* = 300) evaluated pesticide metabolites excreted in the urine within the group, finding significantly lower levels of pesticide metabolites among organic consumers versus conventional consumers, with median concentration levels of investigated metabolites for diethylphosphate (0.196 versus 0.297), dimethylphosphate (0.620 versus 1.382), and total dialkylphosphates (0.12 versus 0.16), *p* < 0.05 [54].

A separate prospective cohort study in adults that estimated organophosphate exposure from food frequency records of 4466 multi-ethnic older Americans, measured urinary pesticide excretion in a sub-group (*n* = 240) and found that higher levels of estimated dietary organophosphate exposure were associated with higher dialkylphosphate concentrations excreted in the urine (*p* < 0.05) [32].

The Million Women Study in the United Kingdom examined any association with cancer incidence and organic diet over a 9-year follow-up period in 1.3 million women. They found no association for reduced cancer incidence in the group, with the exception of a possibly lower incidence of non-Hodgkin lymphoma [65]. 

The Nutri-Net Santé group also investigated associations with cancer incidence in a cohort of 68,946 participants [53]. The group, followed for a mean of 4.6 years, report that after adjustment for confounders, high organic food scores were linearly and negatively associated with the overall risk of cancer (HR for Q4 vs Q1, 0.75; 95%CI, 0.63–0.88; p for trend = 0.001; absolute risk reduction, 0.6%; HR for a 5-point increase, 0.92; 95%CI 0.88–0.96). Amongst specific cancers, they found a decreased risk of developing non-Hodgkin lymphoma (*p* = 0.049) and postmenopausal breast cancer, with no association for other types of cancer. The information on non-Hodgkin lymphoma is similar to that found in the Million Women study; however, the information related to breast cancer was in direct contrast. 

A nested matched case-control study of 300 participants (150 low and 150 high organic food consumers) within the Nutri-Net Santé had serum samples analysed for differences in nutritional biomarkers [20]. No significant differences were found between the 2 groups for α-tocopherol and retinol, cadmium, copper, ferritin or transferrin. Organic consumers exhibited higher plasma concentrations of α-carotene, *β*-carotene, lutein, and zeaxanthin, whereas no differences were found for other carotenoids (*β*-cryptoxanthin and lycopene). Organic consumers had higher levels of magnesium and a lower plasma concentration of iron. Within the fatty acid analysis, organic consumers had lower palmitoleic acid, γ-linolenic acid, and docosapentaenoic acid and higher linoleic acid concentrations. The results of these participants, matched for dietary patterns and other health factors, indicates a possible mild modulation of nutritional levels between organic and non-organic consumers. 

### 3.5. Bias Assessments

The results of bias assessment for cohort studies showed all studies as good or fair, with no studies returning an assessment of poor. Cross-sectional studies were assessed as having a low risk of bias, with the exception of Jensen et al. (1996), which was a short report, with high bias due to missing detail. Within the clinical trials reviewed, the risk of bias was classified as high in several areas, specifically those related to blinding and allocation concealment. Due to the nature of the intervention, in some cases, it was difficult to adequately blind participants (i.e., food packaging, replacement of ‘usual’ diet products). There were, however, several studies [37,38,39,40,41] where blinding and randomisation is stated, but the method is not adequately reported and, therefore, they have received an unclear risk of bias in these areas. Many of the studies were not randomised, providing one diet followed by the alternate diet for all participants concurrently.

Significant bias likely to affect the outcomes of the reports was found for two studies conducted by the same research group in Italy [45,46]. In both cases, all participants received a controlled Mediterranean diet (MD) for 14 days followed by the same diet for a further 14 days using organic rather than conventional foodstuffs, with no washout between diet arms. This introduces a significant risk of bias for the validity of the outcomes for the organic diet intervention as it may be a cumulative effect of the MD changes, rather than a specific effect for the organic component of the diet. 

Another study with high risk of bias was the study by Goen et al. [49] as it contained only two people in the treatment group, in an open-label crossover trial, with no washout between diets. Results of bias assessments are shown in Supplementary Figure S2.

### 3.6. Quality of included Reviews

No formal grading system was applied to the included articles; however, elements of study quality, including high risk of bias or un-realistic results have been discussed for individual articles throughout the review. Several included articles in this present review were not accepted in the previous systematic review into this topic conducted by Dangour et al. (2010). These include pesticide excretion studies [33,72] and a cross-sectional study on semen analyses [51], excluded on the basis of being contaminant studies; and a second semen analysis study [73], excluded as an occupational health study. The rationale for our inclusion of these studies is that although occupational exposure may have been a factor in the Larsen study [73], the method of calculating pesticide exposure was based entirely on food intake. Pesticide excretion studies were included as this was considered potentially important for health, and these studies are also included in other reviews discussing comparison of organic and conventional food intakes on health, i.e., Smith-Spangler et al. [19]. 

## 4. Discussion

This systematic review reports on a wide range of interventional (15 publications) and observational studies (20 publications/13 cohorts), where the health effects of organic diet consumption (whole diet or partial replacement) are compared to conventional diet consumption. Substantially more papers are included compared to previous systematic reviews on this topic [19,35] with varying levels of bias and quality.

### 4.1. Clinical Trials

The included clinical trials use a diverse range of methodologies, all involving short-term food substitutions. These range from acute intake of a single dietary item (conventional or organic), to entire diet substitution over a maximum exposure time of 4 weeks, with most of the studies utilising a 2-week intervention period. The majority of the results show no, or minimal, significant differences between organic (O) and non-organic (NO) treatments in the biomarkers selected. In several of these trials, a single food or drink [37,38,39,40,42,47,48] was substituted for their organic equivalent. Those studies that also compared the composition of the two food items found there was no difference in the concentration of the nutrient of interest (i.e., lycopene) between O and NO foods [37,38,47]. It seems logical, therefore, that a change in participants’ samples would seem unlikely unless there was positive laboratory evidence to demonstrate a specific difference between the NO and O substance that could lead to a biologically plausible difference in vivo. 

Similarly, in whole-diet substitution studies, those that examined antioxidant capacity or nutrients in biomarkers, generally did not show between-group differences, which again appeared to be reflective of the laboratory values of these nutrients were measured [41,47]. However, one study did show a significant change in antioxidant capacity [45]. This study, and a related trial [46], which was the only trial to assess a direct health outcome, both provided a NO Mediterranean diet intervention for 2 weeks prior to 2 weeks of the same O Mediterranean diet. There are several issues with the methodology of this model, these and the associated high risk of bias are discussed further in Section 3.5. The reported weight loss and body composition changes in this study appear unrealistic for the 14-day time frame. The authors report a mean weight loss of 5.6 kg, with mean (SD) weight change from the end of NO diet to end of O diet was 85.17 (±13.97) to 79.52 (±10.41), *p* = 0.0365. The fat loss is reported as 7.18 kg over the two week period from 23.36 (±8.88) to 16.18 (±3.34), *p* = 0.0054, there was also a non-significant 1.18 kg rise in lean muscle mass, from 53.45 (±6.69) to 54.63 (±6.76) [46]. Without baseline assessments provided before any dietary intervention in this group, the effect of the organic intervention cannot be relied upon. 

Whole-diet substitution trials that measured changes in pesticide excretion showed significant and substantial reductions during the O diet phase [31,34,43,44,49], and are discussed under Section 4.3. 

To date, there are no long-term clinical trials measuring direct health outcomes from organic diet intervention. The short timeframe of currently available clinical trials is a serious limitation in assessing demonstrable health benefits. Additionally, only surrogate markers of health have been applied to the majority of clinical trials, with most trials measuring antioxidant levels or pesticide metabolite excretion. 

### 4.2. Observational Research

Observational research, which has followed cohorts for up to 10 years (Nutri-Net Santé and the Million Women study), has investigated a range of hypotheses regarding organic diet and health. Studies included in this review report positive associations between organic diet consumption and a range of areas, including fertility, birth defects, allergic sensitisation, non-Hodgkin lymphoma and metabolic syndrome. 

Findings from two cross-sectional reports on semen parameters detailed mixed findings, and although the majority of tested parameters showed no significant differences, higher sperm concentration in O consumers [50] and lower normal sperm in NO consumers [51] offer preliminary data that is worthy of further exploration. In female fertility, very positive associations between low dietary pesticide exposure and successful pregnancy and birth outcomes in women undergoing assisted reproduction have been reported in one study [52]. Given the declining fertility rates and poorer semen quality being reported worldwide [74], higher odds of achieving clinical pregnancy and live birth with an organic diet is a significant and important finding. A reduction in risk of birth defects (hypospadias) [55,57], but not cryptorchidism [55], and reduced risk of pre-eclampsia [56] add further evidence for organic diet use through pregnancy. 

In children, increased risk of recurrent otitis media has been positively associated with pesticide intake [62], and decreased allergic sensitisation was shown in families following an anthroposophical lifestyle, in comparison to a conventional cohort in the Assessment of Lifestyle and Allergic Disease During Infancy (ALLADIN) study [61]. Consumption of organic dairy products was associated with lower eczema risk as the only significant positive outcome in a similar study (KOALA) [60,70,71]. There are other studies that have supported lower rates of allergic sensitisation from an anthroposophical lifestyle; however, the contribution of organic foods in these studies was not sufficient for them to be included in this review [75,76,77]. Specific confounding factors related to anthroposophic studies are discussed in Section 4.4.

The largest studies reporting on adult populations include the Nutri-Net Santé Cohort Study (France), and the Million Women Study (UK). Both of these studies have investigated associations with cancer risk [64,65], with both finding reduced risk of developing non-Hodgkin lymphoma with increased organic consumption. Other findings between the two studies were similar, with a very small risk reduction (0.6%) for all cancers in France, but no risk reduction in the UK. Postmenopausal breast cancer rates were decreased in high-O consumers [64], but overall breast cancer risk slightly increased in the alternate study [65]. Different adjustment variables between the studies may have been partly responsible for the different outcomes reported, i.e., the Million Women Study adjusted for hormone replacement in breast cancer, which the Nutri-Net Santé study did not report. 

Other findings from the Nutri-Net Santé study show reductions in overweight and risk of obesity, as well as reduced incidence of metabolic syndrome demonstrated in favor of organic food intake [63,64]. Whilst this was self-reported data, there is evidence from other association studies that supports dysregulation of several key facets involved in metabolic syndrome in association with serum pesticides [78,79].

As with any observational studies, there is difficulty in determining the causality of the associations that have been observed. It is possible that the benefits of organic diets are associated only with long-term consumption, or result from lifestyle factors or dietary patterns, which is much harder to model in prospective clinical trials.

### 4.3. Pesticide Excretion

One of the major benefits proposed for organic food is the reduction in exposure to chemicals such as pesticides. Pesticide residues are found in differing amounts across predominantly, fruits and vegetables, but also, grain and dairy products, with much lower amounts found in animal products (except liver, which contains high levels) [24]. 

The major class of pesticides tested for in the organic food literature reviewed for this paper were the organophosphates, the metabolites of which can be measured in the urine as markers of recent exposure. The most commonly detected metabolites are dimethylphosphate, dimethylthiophosphate, diethylphosphate, and diethylthiophosphate. In some studies, herbicide exposure was also assessed, mainly glyphosate, often assessed through its metabolite aminomethylphosphonic acid. Interventions with organic diets markedly reduced the levels of these compounds, and observational studies in adults and children also show reduced urinary metabolite levels in organic versus conventional diets.

Given that several organophosphorus (OP) insecticides and glyphosate (an OP herbicide and the world’s most widely used agricultural chemical) were recently re-classified by the WHO’s International Agency for Research on Cancer (IARC) as being “probably carcinogenic” [80], reduced exposure may potentially benefit health. Results of recent reviews comparing pesticide residues in organic and conventional foods conclude that organic food consumption is one approach to substantially minimise exposure to pesticides [17,21]. 

The impact of switching to organic food consumption on reducing dietary pesticide exposure may be higher in consumers that follow current dietary guidelines for wholegrain and fruit/vegetable consumption. Foods may also be ‘pesticide-free’ but not ‘organic’. It is well documented that pesticide concentrations in wholegrain and wholemeal products are higher than in polished grains such as white flour products (since the outer bran layers of grains have higher pesticide loads then the endosperm) [81]. Apart from wholegrain products, fruits and vegetables are the main dietary source for pesticide exposure and recent European monitoring showed that multiple residues and concentrations above the MRL are most frequently found in fruit and vegetables [24]. 

### 4.4. Confounders of Results

Lifestyle factors amongst organic consumers are likely to have an important impact on external validity. Organic consumers tend to be more health conscious, are more likely to be vegetarian or vegan and are more likely to be physically active [7,8]. 

Epidemiological research has shown consumers of organic food generally have a diet that is higher in plant-based food, lower in animal products, with a higher intake of legumes, nuts, and wholegrains than their conventional food-consuming counterparts. These dietary patterns are likely to have significant health benefits in comparison to what is commonly recognised as the standard Western diet, a diet categorised by highly refined, low-fibre, omnivorous diets low in fruits, vegetables and other plant-based foods [82]. A wholefood diet (high in fibre and plant matter) also has demonstrable effects on a healthy diverse microbiota, which is linked to overall health [83]. The organic consumer group may, therefore, not be representative of the general population, i.e., any benefits from organic food consumption may be attributable partly to increased wholefood intake and a healthier lifestyle.

Whole diet composition and diet quality have been measured and adjusted for in different ways in observational research, with varying elements of the diet included as part of the ‘organic intake’ data collected. It is possible that the benefit observed for organic intake may be partly due to the quality and composition of the diet rather than a direct effect of organic food consumption. Additionally, validation of self-reported organic intake in observational studies is lacking. 

The included cohorts from anthroposophical backgrounds (ALLADIN and KOALA birth cohorts) adds an additional layer of confounding, as the consumption of organic food forms only a small part of the dietary measures adopted in this group. Anthroposophy includes a strong focus on fermented foods, biodynamic production, use of butter and olive oil as predominant fats, and long-term breastfeeding [60,61]. This is combined with other factors such as reduced levels of antibiotic and medication use and a high proportion of plant foods, which together may impact on the overall health of mothers and babies, and influence the results shown.

### 4.5. Limitations

In the included studies, there was wide heterogeneity in the definition and application of the term ‘organic’ and the percentage of organic food replacement in the diet. This makes any interpretation on the benefits or otherwise of organic food consumption very difficult. No formal grading system was applied to the included studies. A grading criteria, such as that employed by Dangour et al. (2010), would have been helpful to categorise the research according to quality. The review was limited by the non-inclusion of foreign language databases.

## 5. Conclusions

A growing number of important findings are being reported from observational research linking demonstrable health benefits to levels of organic food consumption. Clinical trial research has been short-term and measured largely surrogate markers with limited positive results. 

Pesticide excretion studies have consistently shown a reduction in urinary pesticide metabolites with an organic diet; however, there is insufficient evidence to show translation into clinically relevant and meaningful health outcomes. There is a need for studies to move beyond simply measuring the reduction in pesticide exposure with organic food, to investigating measurable health benefits. 

The finding that organic food consumption substantially reduces urinary OP levels is important information for consumers, who would like to take a precautionary approach and minimise OP-pesticide exposure. Given the current knowledge on the toxicity of these chemicals, it seems possible that ongoing reduced exposure may translate to health benefits. 

While findings from this systematic review showed significant positive outcomes from observational studies in several areas, including reduced incidence of metabolic syndrome, high BMI, non-Hodgkin lymphoma, infertility, birth defects, allergic sensitisation, otitis media and pre-eclampsia, the current evidence base does not allow a definitive statement on the long-term health benefits of organic dietary intake. Consumption of organic food is often tied to overall healthier dietary practices and lower levels of overweight and obesity, which are likely to be influential in the results of observational research.

### Recommendations for Future Research

Single-food substitution studies have shown no benefits and should not be undertaken without substantive pre-clinical data. Additionally, surrogate markers, i.e., antioxidant levels and pesticide excretion, are insufficient to determine actual benefit to health and ideally should be coupled with measurements related to specific health outcomes. Unlike the current exposure studies which measure changes in days or weeks, longer-term health benefit studies are needed. Specifically, long-term whole-diet substitution studies, using certified organic interventions will provide the most reliable evidence to answer the question of whether an organic diet provides true measurable health benefits.

Additional research options may include further evaluation of biological data collected through previous large cohort studies, such as the Nutri-Net Santé study [84], and the MoBa biobank [85], to test hypotheses on organic diet and health.

## Figures and Tables

**Table 1:** Data extraction table—Clinical trials.

Ref.	Study population	*n*	Design and Duration	Exposure/treatment	Outcome measures	Results	Definition of organic
Caris-Veyrant (2004) [37]	France Age: 21–39 years 100% female Healthy	24	Parallel RCT. Single-blind. 2-arm, 3 week dietary intervention.	100 g of conventional tomato puree (NO), or organic tomato puree (O) added to lunch or dinner once daily.	Plasma Vit C, *β*-carotene and lycopene.	Tomato puree increased plasma *β*-carotene and lycopene in both groups. There were no significant between-group differences in any outcome. The two purees had similar lycopene and beta-carotene contents.	Describes growing conditions of both crops. They were both experimental crops for the study.
Stracke (2009) [38]	Germany Age: 19–54 years 100% male Healthy	36	Parallel RCT. Double-blind. 3-arm, 14 day intervention, with 4-week low carotenoid diet prior to baseline.	200 g of conventional blanched carrots (NO) or organic blanched carrots (O) consumed with main meal and minimum 10 g fat. Control (C) followed carotenoid restricted diet.	Plasma carotenoid concentration (α- and β-carotene, lycopene, lutein, zeaxanthin and b-cryptoxanthin), Vit E, Vit C, antioxidant activity (FRAP, ORAC, TEAC), and LDL oxidation; cytokine quantity, NK cell quantity and activity, DNA damage, plasma glucose, uric acid, TAG, cholesterol.	No significant difference in any outcome measure. There was no significant difference in carotenoid concentration of O and NO carrots.	The fertilisation, harvest and distribution of the carrots were monitored by the Institute of Organic Farming of the Johann Heinrich von Thünen Institute, Federal Research Institute for Rural Areas, Forestry and Fisheries at Westerau.
Stracke (2010) a) [39]	Germany Study 1: Age: 23–32 years 100% male Healthy	6	Crossover. Double-blind RCT. 2 phases, single consumption after overnight fast.	1000 g of conventional apples (NO) or organic apples (O)	Apple polyphenols and their metabolites, total antioxidant status (FRAP, TEAC, and ORAC).	There were no significant differences between the O and NO intake in any of the analysed polyphenols. Apple consumption had no effect on TEAC, ORAC or FRAP.	Cultivated according to the requirements of “Bio Suisse” (predominant label organisation for certified organic production in Switzerland).
Stracke (2010) b) [39]	Germany Study 2: Age: (22–40yrs) 100% male Healthy	43	Parallel RCT Double-blind. 3-arm, 5-week study (1-week depletion period /4-week intervention).	500 g of conventional apples (NO) or organic apples (O). The third group served as control group (C), and maintained an apple- and polyphenol restricted diet.	Apple polyphenols and their metabolites, glucose, TAG, cholesterol, WBC, and uric acid; total antioxidant status (FRAP, TEAC, and ORAC); Vit C, Vit E, carotenoids.	No between group differences between O and NO groups in plasma glucose, uric acid, TAG, cholesterol, Vit C, Vit E, carotenoids, WBC, polyphenol concentrations, or antioxidant markers.	Cultivated according to the requirements of “Bio Suisse” (predominant label organisation for certified organic production in Switzerland).
Briviba (2007) [40]	Germany Age: 23–32 years 100% male Healthy	6	Crossover. Double-blind RCT. 2 phases. 3-day polyphenol depletion prior to single consumption (after overnight fast), 1-week washout.	Single consumption of 1000 g of conventional apples (NO) or organic apples (O).	Antioxidant activity, LDL oxidation, DNA damage (comet assay).	There were no statistically significant differences between groups on DNA damage, antioxidant activity or LDL oxidation.	Cultivated according to the requirements of “Bio Suisse” (predominant label organisation for certified organic production in Switzerland).
Grinder-Pedersen (2003) [41]	Denmark Age: 21–35 years 6 males + 10 females Healthy	16	Crossover. Double-blind RCT. 2 phases. 1 week run-in (excluding flavonoid-containing foods), 22 days each intervention with 3-week washout.	Whole diet intervention. Two intervention diets: conventional (NO) and organic (O); consisted of 4 different menus with identical meals and quantities.	SOD, Gpx, GR, Cat, TEAC, FRAP, malondialdehyde, 22-AAS. 24hr urine samples (Days 0 and 22): measured flavonoids (quercetin, kaempferol, and isorhamnetin) and flavonones (naringenin and hesperitin).	Quercetin (*p* < 0.01) and 2-AAS (*p* < 0.05) were significantly higher, as was urinary excretion of quercetin and kaempferol (*p* < 0.05) in O phase. TEAC was significantly increased (*p* < 0.05) after intake of NO compared to O. No significant difference was seen for isorhamnetin (*p* = 0.07) or kaempferol (*p* = 0.10).	No organic certification defined. The study used local, known conventional and organic growers (pork - from same litter, dairy, eggs, fruit and vegetables sowed and harvested within same week and from similar geographic location).
Akcay (2004) [42]	Turkey Age: 24–54 years 6 male + 2 female Healthy	8	Crossover trial. 2 phases. 6-week washout.	Single dose of conventional wine (NO), or organic wine (O). Men drank 200 mL (alcohol content 24 g) and women drank 100 mL (alcohol content 12 g) over 15 minutes.	Blood samples at 0, 60 and 360 minutes, measured total phenol content, SOD, Cat, TBARS, LDL-TBARS.	Very poorly reported results. SOD increased at 1 hour (*p* = 0.046) and 6 hours (*p* = 0.028) in O group compared to baseline (no group comparison). SOD at 6 hour increased in NO group compared to baseline (no values given). No significant difference in TBARS.	No organic certification defined. "The organic wine Cabernet Sauvignon (CS) was obtained by defined standards (certificated grapes of *Vitis vinifera* origin..”
Lu (2006) [43]	United States Age: 3–11 years 13 male + 10 female School children	23	Crossover trial. 3 phases: conventional diet (NO) days 1–3 and days 9–15, organic diet (O) days 4–8.	Food items were substituted for most of children’s conventional diet, including fruits, vegetables and grains for 5 days. Urine samples (first and last of the day) collected for whole 15 day period.	Metabolites for selected OP pesticides, pyrethroid insecticides, and herbicides.	Immediately after beginning O diet, median urinary MDA and TCPY decreased to non-detectable levels, where they remained until conventional diets were reintroduced (*p* < 0.01). No effect of diets on other metabolites.	No organic certification defined. "All organic food items were purchased by the research staff from a single grocery store.”
Lu (2008) [44]	United States Age: 3–11 years 13 male + 10 female School children	23	Crossover trial. 3 phases: conventional diet (NO) days 1–3 and days 9–15, organic diet (O) days 4–8.	Details as above, including testing for 15- or 12-consecutive-days in the summer (July–August) and fall (October–November), respectively, and a 7-consecutive-day sampling period in both the winter (January–February) and spring (April–May).	Metabolites for selected OP pesticides, pyrethroid insecticides, and herbicides.	Authors observed a seasonal effect on organophosphorus urinary biomarker levels in this cohort, and this seasonality corresponds to the consumption of fresh produce among the children throughout the year. This study is extended seasonal data for the same study detailed in Lu 2006.	No organic certification defined. "All organic food items were purchased by the research staff from a single grocery store.”
Di Renzo (2007) [45]	Italy Age: 30–65 years 100% male Healthy	10	Crossover trial. 2 phases. 14 days diet 1, then 14 days diet 2 - no washout.	Conventional Mediterranean diet intervention (NO), followed by organic Mediterranean diet intervention (O).	Plasma antioxidant (ORAC) capacity.	ORAC after NO Mediterranean diet was 2.25 mM TE, and 2.75 mM TE after O Mediterranean diet. This was a significant increase (21%) after the consumption of O diet.	No organic certification defined. Described as an "exclusively organic" diet for the organic treatment arm.
De Lorenzo (2010) [46]	Italy Age: 30–65 years 100% male *n*=100 healthy; *n*=50 stable chronic kidney disease (CKD)	150	Crossover trial. 2 phases. 14 days diet 1, then 14 days diet 2 - no washout.	Organic Mediterranean diet intervention (NO), followed by organic Mediterranean diet intervention (O).	BMI, DXA, Hcy, serum phosphorus, blood glucose concentrations, lipid profile, inflammatory markers, microalbuminuria.	DXA showed significant differences between NO Mediterranean diet and O Mediterranean diet for fat mass (*p* < 0.001), average loss of 6.1 kg. Significant decrease in cholesterol (*p* = 0.04), calcium and microalbuminuria (*p* = 0.003) after O diet only in CKD patients. Inflammatory parameters decreased in both groups after the O diet.	No organic certification defined. Described as an "exclusively organic" diet for the organic treatment arm.
Soltoft (2011) [47]	Denmark Age: 18–40 years 100% male Healthy	18	Crossover. Double-blind RCT. 3 phases. 12 days each intervention with 2 week wash-out.	3 x treatment arms. (OA: organic based on livestock manure, OB: organic based on green manure and NO: conventional with mineral fertilizers) grown in two consecutive years (year 1 and 2). Diets fully controlled.	Fasting blood samples (day 1 and day 13 of each treatment arm) analysed for carotenoid content.	There was no significant difference in the plasma carotenoid content from the three different diets. There was very little difference between the concentrations of carotenoids in the carrots across growth systems, or across year to year of crops.	The organic growth systems were managed in compliance with the Danish guidelines for organic farming administered by the Danish Plant Directorate.
Toaldo (2016) [48]	Brazil Age: 20–55 years 15 male + 28 female Healthy	24	Crossover. Single-blind RCT. 3 phases. 14-day washout. 3 days polyphenol depletion prior to acute dose.	3 x treatment arms. Single dose of 400 mL of conventional juice (NO), organic juice (O), or water. Blood samples were collected at 0 and 60 minutes.	GSH, Cat, SOD, Gpx, TAC, glucose, and uric acid.	GSH increased by 8.2% (*p* < 0.001) and 7.0% (*p* < 0.05) after NO and O, respectively, with no significant difference between juices. CAT increased 22% after O (*p* < 0.001). SOD increased 12.9% and 16.3% after NO and O, respectively (*p* < 0.001). GPx increased 6.9% and 7.3%, respectively, after NO and O (*p* < 0.05).	No organic certification defined. "Two red grape juices were used in this study: an organic juice prepared with organic Bordo grapes and a conventional juice prepared with conventional grapes.."
Goen (2017) [49]	Switzerland Age: 46–49 years 1 male + 1 female Healthy	2	Crossover trial. 2 phases. 11 days on conventional diet, followed by 18 days organic diet, no washout.	Conventional diet (NO) or organic diet (O). Participants purchased/ prepared all food. Urine samples taken for last 4 days of each intervention.	Urinary pesticide excretion, including DAP, pyrethroid metabolites, chlorinated phenoxycarboxylic acids, glyphosate, AMPA.	This very small study (*n* = 2) shows some small but statistically significant reduction in some components of pesticide exposure with O diet. NO shows organophosphate pesticides and some chlorinated phenoxy carboxylic acids as main exposure components.	Not defined. "participants switched to exclusively organic food intake"
Bradman (2015) [31]	United States Age: 3–6 years 19 male + 21 female Pre-school children	40	Crossover trial. 3-phases: conventional diet (NO) days 1–4, organic diet (O) days 5–11, conventional diet (NO) days 12–16.	Prior to study children enrolled primarily consumed conventional diet. Urine samples collected over 16 consecutive days. Food diaries kept during study phases.	Urinary concentrations of pesticides (23 pesticide metabolites including specific and nonspecific metabolites for OP, pyrethrin, and pyrethroid insecticides and select herbicides).	Most metabolites were below LOD, mean concentrations of 6 were lower during O for all children, and were significant for total DAPs and dimethyl DAPs and 2,4-D (2,4-dichlorophenoxyacetic acid, a herbicide), with reductions of 40%, 49%, and 25%, respectively (*p* < 0.01).	No organic certification defined. Food for the organic phase was provided by the researchers according to the families shopping list request (to maintain diet similarity).
Oates (2014) [34]	Australia Age: mean 42 years 4 male + 9 female Healthy	13	Crossover. RCT. 2 phases: conventional diet (NO) or ≥80% organic diet (O). 7 days per intervention, no washout.	Participants maintained usual dietary choices and sourced own food. Spot morning urine sample analysed on day 8 of each diet.	Urinary concentrations of pesticides, including six DAP metabolites of OP pesticides (DMP, DMTP, DMDTP, DEP, DETP and DEDTP).	Statistically significant lower levels of urinary DMP and DMTP (*p* < 0.05), with a trend for DMDTP during O phase. No significant difference for DEP, DETP, and DEDTP. Overall pesticide results in the O phase were 89% lower than in NO phase (*p* = 0.013).	Not defined. "Participants were asked to consume as close to 100% conventional or organic food as possible during each 7 day dietary period."

Abbreviations: 2-AAS: 2-amino-adipic semialdehyde; AMPA: aminomethylphosphonic acid; BMI: body mass index; C: control group; CKD: chronic kidney disease; Cat: catalase; CS: cabernet sauvignon; DAP: dialkylphosphate; DEP: diethylphosphate; DETP: diethylthiophosphate; DEDTP: diethyldithiophosphate; DMDTP: dimethyldithiophosphate; DMP: dimethylphosphate; DMTP: dimethylthiophosphate; DNA: deoxyribonucleic acid; DXA: dual-energy X-ray absorptiometry; FRAP: ferric reducing ability of plasma; GPx: glutathione peroxidase; GR: glutathione reductase; GSH: glutathione; Hcy: homocysteine; LDL: low density lipoprotein; MDA: malathion; NK: natural killer; NO: non-organic group; O: organic group; OP: organophosphate; ORAC: oxygen radical absorbance capacity; RCT: randomised controlled trial; SOD: superoxide dismutase; TAC: total antioxidant capacity; TAG: triacyglycerol; TBARS: thiobarbituric acid reactive substances; TCPy: 3,5,6-trichloro-2-pyridinol; TE: trolox equivalents; TEAC: trolox equivalents antioxidant capacity; Vit: vitamin; WBC: white blood cell.

**Table 2:** Data extraction table - Observational Studies.

Ref	Study Population	*n*	Design and Duration	Exposure/Treatment	Outcome Measures	Results	Definition of Organic
Jensen (1996) [50]	Denmark Age: mean 33 years 100% male Members of organic farming organisations (*n* = 55) / airline company (*n* = 141)	196	Cross-sectional study. Analysis of semen samples for sperm quality in male organic farmers and airline workers.	Diet, working conditions, health, and lifestyle were assessed with questionnaire. Those with >25% organic diet defined as organic group. Self-reported FFQ.	Comparison of sperm concentration, seminal volume, total sperm count, and sperm morphology.	Sperm concentration was 43.1% (95%CI 3.2%–98.8%, *p* = 0.033) higher among men eating organically produced food. Seminal volume, total sperm count, and sperm morphology were not different between groups. This was a short report and missing detail on organic diet definitions between groups.	No specific definition of organic.
Juhler (1999) [51]	Denmark Age: mean 38 years 100% male Organic farmers (*n* = 85) / conventional farmers (*n* = 171)	256	Cross-sectional study. Analysis of semen samples for sperm quality in organic vs. conventional farmers.	Farmers divided into three groups, according to organic production/proportion of organic food consumption: none (N, 0%), medium (M, 1–49%), or a high (H, 50–100%) proportion FV consumed. Self-reported FFQ.	Correlation between estimated dietary pesticide intakes and semen parameters (including sperm concentration, seminal volume, total sperm count, and sperm morphology).	Group N showed a significantly lower proportion of morphologically normal spermatozoa, but no difference in 14 other semen parameters. A higher intake of five specific pesticides equated with a lower percentage of dead spermatozoa. No other significant differences were found.	No specific definition of organic.
Chiu (2018) [52]	United States; Environment and Reproductive Health (EARTH) Study Age: mean 35 years 100% female Women attending fertility clinic	325	Prospective cohort. Artificially assisted reproduction (AAR) outcomes in women, including pregnancy/birth outcomes associated with high and low dietary pesticide exposure.	Self-reported FFQ, prior to starting AAR. A total Pesticide Residue Burden Score (PRBS) was calculated (based on pesticide residue data and organic FV intake). Classifications were organic >3 times/week, or non-organic <3 times/week.	Clinical outcomes included implantation, clinical pregnancy, live birth. Early ART end points included markers of ovarian responses to stimulation (peak estradiol levels, endometrial thickness, oocyte development, total oocytes), fertilization rate, and embryo quality.	High PRBS was inversely associated with probability of clinical pregnancy and live birth per initiated cycle. Compared with women in the lowest quartile of high-pesticide residue FV intake (<1 serving/day), women in the highest quartile (≥2.3 servings/d) had 18% (95%CI, 5%–30%) lower probability of clinical pregnancy and 26% (95%CI, 13%–37%) lower probability of live birth. No association was found between quartiles and early ART end points. The adjusted probabilities of total pregnancy loss were 7% (95%CI, 3%–15%), 23% (95%CI, 16%–33%), 24% (95% CI, 15%–36%), and 34% (95% CI, 20%–51%) for women in increasing quartiles of high–pesticide residue FV intake.	No specific definition of organic. Volunteers were asked to provide information on frequency of organic FV consumption (<3 vs ≥3 times/week).
Baudry (2018) [53]	France; Nutri-Net Santé Cohort study Age: mean 44 years 78% female General population	68,946	Prospective observational cohort study (internet-based). Followed for up to 7 years, looking at all first primary cancers diagnosed between study inclusion and November 2016.	FFQ and cancer data (self-reported, but verified with medical records in >90% of cases). Estimated intake of 16 organic food/beverage items recorded to determine an organic score. Organic quartiles: Q4 = highest organic food intake, Q1 = lowest organic food intake.	All first primary cancers diagnosed between study inclusion and November 2016. All cancer types considered cases except for basal cell skin carcinoma, which was not considered cancer.	High organic food scores were linearly and negatively associated with the overall risk of cancer (HR for Q4 vs Q1, 0.75; 95%CI, 0.63–0.88; P for trend = .001; absolute risk reduction, 0.6%; HR for a 5-point increase, 0.92; 95% CI, 0.88–0.96). Within individual cancer types, a significantly reduced HR was seen for those with Q4 intake vs. Q1 for all lymphomas, non-Hodgkin lymphoma and post-menopausal breast cancer.	No specific definition of organic. Consumption frequency of 264 food and drink items used to calculate organic score.
Baudry (2018) [20]	France; Nutri-Net Santé Cohort study Age: mean 58 years 70% female General population	300	Nested matched case-control study of 300 participants (150 low and 150 high organic food consumers), with available fasting blood samples for analysis.	Self-reported FFQ used to estimate organic food intake. Low and high organic food consumers were grouped according to proportion of organic food below 10% or above 50%. The average proportions of organic food in the diet were 3% (± 3) and 67% (± 13) in the conventional and organic groups, respectively.	Plasma concentrations of vitamins A and E as well as 6 carotenoids (α-carotene, *β*-carotene, *β*-cryptoxanthin, lutein, zeaxanthin, and lycopene), copper, cadmium, magnesium, iron, transferrin and ferritin, fatty acid composition.	No significant differences were found between the 2 groups for α-tocopherol and retinol, cadmium, copper, ferritin or transferrin. Organic consumers exhibited higher plasma concentrations of α-carotene, *β*-carotene, lutein, and zeaxanthin with no differences for other carotenoids. Organic consumers had higher magnesium, lower iron, lower palmitoleic acid, γ-linolenic acid, and docosapentaenoic acid, and higher linoleic acid.	No specific definition of organic. Consumption frequency of 264 food and drink items used to calculate organic score.
Baudry (2019) [54]	France; Nutri-Net Santé Cohort study Age: mean 58 years 70% female General population	300	Nested matched case-control study of 300 participants (150 low and 150 high organic food consumers), with available urine samples for analysis.	Self-reported FFQ used to estimate organic food intake. Low and high organic food consumers were grouped according to proportion of organic food below 10% or above 50%. The average proportions of organic food in the diet were 3% (± 3) and 67% (± 13) in the conventional and organic groups, respectively.	Urinary pesticide and metabolite concentrations (organophosphorus, pyrethroid, and azole compounds).	Pesticide concentrations were mostly below LOD. For pesticide metabolites, significantly higher levels of DETP, DMTP, total DAPs (organophosphorus metabolites) and free 3-PBA (a pyrethroid metabolite) were found among conventional consumers compared to organic consumers, with median concentration levels of diethylphosphate (0.196 versus 0.297), dimethylphosphate (0.620 versus 1.382), and total dialkylphosphates (0.12 versus 0.16), *p* < 0.05.	No specific definition of organic. Consumption frequency of 264 food and drink items used to calculate organic score.
Brantsæter (2016) [55]	Norway; The Norwegian Mother and Child Cohort Study (MoBa). 100% female Pregnant women who delivered a singleton male infant.	35,107	Prospective cohort. Pregnant women at gestational week 22 surveyed for organic food consumption with results correlated to prevalence of male infants born with hypospadias or cryptorchidism.	Self-reported FFQ collected information about average dietary intake since start of pregnancy over six groups of organically produced food (vegetables, fruit, bread/cereal, milk/dairy products, eggs, and meat).	Association between non-organic/organic food consumption (never/seldom vs sometimes/often/mostly) and development of hypospadias or cryptorchidism in male newborns.	Seventy-four male newborns were diagnosed with hypospadias (0.2%), and 151 with cryptorchidism (0.4%). Women who consumed any organic food during pregnancy were less likely to give birth to a boy with hypospadias (OR = 0.42; 95% CI: 0.25, 0.70, based on 21 exposed cases) than women who reported they never or seldom consumed organic food. Associations with specific organic foods were strongest for vegetable (OR = 0.36; 95% CI: 0.15, 0.85; 10 exposed cases) and milk/dairy (OR = 0.43; 95% CI: 0.17, 1.07; 7 exposed cases) consumption. No association was observed for consumption of organic food and cryptorchidism.	All food sold as organic in Norway must be certified by Debio. Debio is accredited organic by Norwegian Accreditation and by IFOAM.
Torjusen (2016) [56]	Norway; The Norwegian Mother and Child Cohort Study (MoBa) Age: mean 28 years 100% female Nulliparous pregnant females.	28,192	Prospective cohort. Pregnant women at gestational week 22 surveyed for organic food consumption with results correlated to prevalence of pre-eclampsia.	Among the 28,192 women in this study, the majority reported never/rarely eating organic food; 39.8% ate at least one organic food ‘sometimes’; 7% ate at least one organic food ‘often’; and 1.8% reported use of any organic food ‘mostly’.	Pre-eclampsia in pregnant women.	The prevalence of pre-eclampsia in the study sample was 5.3% (*n* = 1,491). Women who reported eating organic ‘often’ or ‘mostly’ (*n* = 2,493, 8.8%) had lower risk of pre-eclampsia than those who reported ‘never/rarely’ or ‘sometimes’ (crude OR = 0.76, 95%CI 0.61, 0.96; adjusted OR = 0.79, 95%CI 0.62, 0.99). The lower risk was evident also when adjusting for overall dietary quality.	No specific definition of organic. Frequent organic consumption was defined as eating organic food ‘often’ for at least one of the six food categories.
Christensen (2013) [57]	Denmark 100% female Mothers of boys operated on for hypospadias (*n* = 306) and matching control group (*n* = 306)	612	Retrospective case-control study. Retrospective interviews of organic dietary habits in mothers with male infant born with hypospadias and matched controls.	FFQ listed choice of organic food items in the first trimester for milk, other dairy, eggs, meat, FV. Responses consisted of often, sometimes, rarely and never. Current dietary habits (up to several years post-pregnancy) were taken as proxy for pregnancy diet.	Association between organic food consumption of specified food groups during pregnancy and prevalence of hypospadias in infant sons.	Higher OR for hypospadias was found with rare or no consumption of organic non-milk dairy products, however, the association was not statistically significant after adjustment (OR = 1.36, 95%CI 0.95, 1.94). A similar association was observed for mothers rarely or never choosing organic eggs (OR = 1.28, 95%CI 0.92, 1.79). Total organic intake showed no statistically significant association, however, mothers who never or rarely chose any organic products had nonsignificant increased odds of giving birth to a boy with hypospadias (adjusted OR = 1.31, 95%CI 0.78, 2.21).	No specific definition of organic.
Rist (2007) [58]	Netherlands; KOALA Birth Cohort Age: mean 33 years 100% female Breastfeeding mothers with conventional (NO) or alternative (O) lifestyle	312	Cross-sectional study. Analysis of breast milk for fatty acid content from lactating women with predominantly organic or non-organic food consumption.	FFQ at gestational week 34. Classification into four groups based on the origin of meat/dairy products only. Organic = >90% organic Moderate = 50-90% organic Conventional = <50% organic	Amount of conjugated linoleic acids in breast milk of lactating women, measured as trans-vaccenic acid (TVA) and cis-9,trans-11-octadecadienoic acid (Rumenic).	Rumenic acid increased in a statistically significant way moving from a conventional diet (*n* = 186) to a moderately organic diet (*n* = 33), to a strict organic diet (*n* = 37). TVA levels were higher in the two mostly organic quartiles than in the conventional or minimal groups.	No specific definition of organic. Food origin specified as conventional or organic and % of food group as <50 %, 50–90% or >90%.
Mueller (2010) [59]	Netherlands; KOALA Birth Cohort Age: mean 33 years 100% female Breastfeeding mothers with conventional (NO) or alternative (O) lifestyle	310	Cross-sectional study. Analysis of breast milk for trans fatty acid content from lactating women with predominantly organic or non-organic food consumption.	FFQ at gestational week 34. Classification into four groups based on the origin of meat/dairy products only. Organic = >90% organic Moderate = 50-90% organic Conventional = <50% organic	Amount of trans fatty acids (TFA) in breast milk of lactating women, measured as different trans fatty acid isomers.	Total TFA content of mothers’ milk in the compared groups ranged between 3 and 3.3% of total fatty acids. There were no significant differences in the total TFA content between groups of organic vs. non-organic intake or amount of dairy fat intake reported.	No specific definition of organic. Food origin specified as conventional or organic and % of food group as <50 %, 50–90% or >90%.
Kummeling (2008) [60]	Netherlands; KOALA birth Cohort Age: 2 years Infants with non-organic (NO) (*n* = 2,135), or organic diet (O) (*n* = 463)	2598	Prospective cohort. Mothers of infants surveyed about child’s organic food consumption and allergy symptoms at 3, 7, 12 and 24 months of age.	Parents completed FFQ at each time-point. Infants diet classified as: ‘conventional’ (<50% organic); ‘moderately organic’ (50–90% organic); ‘strictly organic’ (>90% organic).	Association between allergic symptoms reported by parents (including eczema, wheeze occurrence, rash) and intake of organic vs. conventional foods; IgE antibodies measured in a subset of children (*n* = 815).	Consumption of organic dairy products was associated with lower eczema risk (OR = 0·64, 95%CI 0·44, 0·93), but there was no association for development of eczema, wheeze or atopic sensitisation. No statistically significant associations were observed between organic food consumption and recurrent wheeze (OR = 0·51, 95%CI 0·26, 0·99) during the first 2 years of life.	In the Netherlands ‘organic’ products include biodynamic production, which carry the registered ‘EKO’ certification.
Stenius (2011) [61]	Sweden; ALLADIN Study Age: foetal period (2^nd^ trimester of mother) – 24 months	330	Prospective cohort. Anthroposophic or non-anthroposophic families followed for development of allergic sensitisation in children, correlated with lifestyle factors (including organic food choice).	FFQ completed by pregnant women in 2^nd^ trimester. Child followed for allergic sensitisation to 24 months. Organic food consumption in AL group was 80% and 5% in CL group.	IgE in cord blood and sensitisation to common allergens and total IgE at 6, 12, and 24 months of age.	Children of families with AL had a markedly decreased risk of sensitisation during the first 2 years of life compared with children of CL families with adjusted OR = 0.25 (95%CI 0.10, 0.64), *p* = 0.004. Children from families with a partly anthroposophic lifestyle had similar result with adjusted OR = 0.31 (95%CI 0.15, 0.54), *p* = 0.002.	No specific definition of organic. Organic/biodynamic diet evaluated as one of many lifestyle questions, with no detail of how this was quantified.
Buscail (2015) [62]	France; PELAIGE mother-child cohort Age: foetal period (from gestation) – 24 months	1505	Prospective observational cohort study. Mothers consumption of organic food mid-pregnancy and when infant is 2 years, correlated to episodes of otitis media.	Pregnant women completed questionnaires reporting domestic use of pesticides and consumption of organic diet during pregnancy at 19 weeks of gestation and again at age 2 of infant. Children were assessed for otitis media during early childhood.	Episodes of otitis media (OM) and recurrent OM in children. Urinary samples to measure pesticides (*n* = 248). Associations between pesticide measurements and OM.	Children whose mothers reported an organic diet during pregnancy had a reduced risk of OM (at least one episode, *p* trend = 0.01). No association was found between any outcome and residential proximity to crops. The presence in maternal urine of dealkylated triazine metabolites (herbicide) was positively associated with recurrent OM (OR = 2.12 (1.01 to 4.47)).	No specific definition of organic. Fruit, vegetables and cereals from a non-organic diet were selected as proxies for insecticide exposure.
Kesse-Guyot (2017) [63]	France; Nutri-Net Santé Cohort study Age: mean 45 years 78% Female General population	62,224	Prospective cohort (internet-based). Followed for up to 10 years, looking at body weight change, risk of overweight or obesity and consumption of organic food.	Self-reported FFQ and anthropometric data completed annually (average 3.1 year follow-up). Estimated intake of 16 organic food/beverage items recorded to determine an organic score (OS). Organic diet quartiles: Q4 = highest rate of organic food consumption, Q1 = lowest rate of organic food consumption.	Correlation between the OS and change in BMI during follow-up and risk of overweight and obesity.	Lower BMI increase was observed across quartiles of the OS (mean difference Q4 v.Q1 = −0.16 (95%CI −0.32, −0.01). An increase in the OS was associated with a lower risk of overweight and obesity (among non-overweight and non-obese participants at inclusion): OR for Q4 v. Q1 were 0.77 (95%CI 0.68, 0.86) and 0.69 (95%CI 0.58, 0.82), respectively. The association remained strong and highly significant, with a reduction in the risk of obesity of 37% at follow-up. A similar association was observed for overweight, although the strength of the association was smaller.	No specific definition of organic. Consumption frequency of 264 food and drink items used to calculate organic score.
Baudry (2017) [64]	France; Nutri-Net Santé Cohort study Age: mean 45 years 78% Female General population	8174	Cross-sectional analysis of proportion of organic food in the diet (overall and by food group) and prevalence of metabolic syndrome.	Participants filled out a self-administered FFQ, including 264 food and beverage items. Separated into tertiles of organic food consumption.	Correlation between level of organic food intake and prevalence of metabolic syndrome.	Higher organic food consumption was associated with a lower probability of metabolic syndrome, being negatively associated with prevalence, 0.69 (95%CI 0.61, 0.78) when comparing the third tertile of proportion of organic food in the diet with the first one (*p* < 0.0001).	No specific definition of organic. Consumption frequency of 264 food and drink items used to calculate organic score.
Bradbury (2014) [65]	United Kingdom; The Million Women Study Age: mean 59 years 100% Female General population	623,080	Prospective cohort. Study correlates frequency of organic food intake to cancer incidence in women, followed on average for 9.3 years.	Women without cancer at baseline completed a questionnaire asking ‘Do you eat organic food?’ with four possible responses: ‘never, sometimes, usually, and always.’ Repeated at follow-up (on average 9.3 yr).	Association of organic diet with cancer, including all cancers combined (except non-melanoma skin cancer), oral, oesophageal, stomach, colorectum, pancreas, lung, malignant melanoma, breast, endometrium, ovary, kidney, bladder, brain, non-Hodgkin lymphoma, multiple myeloma, and leukaemia.	At baseline, 30%, 63% and 7% of women reported never, sometimes, or usually/always eating organic food, respectively. Consumption of organic food was not associated with a reduction in the incidence of all cancer (*n* = 53,769 cases in total) (RR for usually/always vs never = 1.03, 95%CI 0.99, 1.07), soft tissue sarcoma (RR = 1.37, 95%CI 0.82, 2.27), or breast cancer (RR = 1.09, 95%CI 1.02, 1.15), but was associated with reduced risk for non-Hodgkin lymphoma (RR = 0.79, 95%CI 0.65, 0.96).	No specific definition of organic.
McGuire (2016) [66]	United States Age: mean 29 years 100% female Breastfeeding women 1–3 months postpartum	41	Cross-sectional study. Single milk and urine sample from each woman to assess level of pesticides.	5 question survey that documented potential glyphosate exposure from environment and diet. 42% of the women identified as having "strictly or mainly organic food choices"	Glyphosate and AMPA concentrations in human milk, correlated with pesticide excretion in urine samples.	Glyphosate and AMPA were not detectable in milk samples. There were no significant effects of consuming organic over conventional foods or living on/near a farm compared with living in an urban/suburban region on concentrations of glyphosate (*p* = 0.1870 and 0.8773, respectively), or AMPA in urine (*p* = 0.1414 and 0.2525, respectively).	No specific definition of organic. Food intake was self-reported as either mainly organic or mainly conventional.
Curl (2003) [33]	United States Age: 2–5 years 56% Male Children	39	Cross-sectional study. Level of organic food (%) in diet correlated to pesticide excretion in urine. Food frequency data and urine samples were collected.	Parents of children interviewed about diet, health information and pesticide use, with 2 day food diary completed on day of child's urine sampling. Diet reported as mostly organic or mostly conventional.	24-hour urine samples measured for urinary DAP or DMP concentrations.	The median total DMP concentration was approximately six times higher for children with conventional diets than for children with organic diets (0.17 and 0.03 µmol/L; *p* = 0.0003); mean concentrations differed by a factor of nine (0.34 and 0.04 µmol/L).	No specific definition of organic. >75% of dietary intake as organic or conventionally separated the two groups.
Curl (2015) [32]	United States Age: 45–84 years 50% Female Adults with subclinical cardiovascular disease	6814	Cross-sectional study. Food frequency data and urine samples were collected, correlating organic intake to urinary excretion of pesticides.	Diet was reported as mostly organic or conventional. Participants were asked about their “usual” intake of specific foods and beverages “over the past year.” Average pesticide intake was then calculated.	Correlation between pesticide intake and excretion of pesticides in urine. Sub-group analysis of urine samples for pesticides (*n* = 240).	Among conventional consumers, increasing tertile of estimated dietary organophosphate exposure was associated with higher DAP concentrations (*p* < 0.05). DAP concentrations were also significantly lower in groups reporting more frequent consumption of organic produce (*p* < 0.02).	No specific definition of organic. Foods eaten are correlated to US Dept Agriculture data on pesticide residues and a pesticide exposure number assigned.

Abbreviations: AAR: artficially assisted reproduction; AL: anthroposophic lifestyle; ART: assisted reproductive technology; AMPA: aminomethylphosphonic acid; BMI: body mass index; CL: conventional lifestyle; DAP: dialkylphosphate; DETP: diethylthiophosphate; DMP: dimethylphosphate; DMTP: dimethylthiophosphate; FFQ: food frequency questionnaire; FV: fruits and vegetables; HR: hazard ratio; LOD: limit of detection; NO: non-organic group; O: organic group; OM: otitis media; OS: organic score; PBA: 3-phenoxybenzoic acid; PRBS: pesticide residue burden score; TFA: trans-fatty acid; TVA: trans-vaccenic acid; Vit: vitamin.

## Supplementary Materials

The following are available online at https://www.mdpi.com/2072-6643/12/1/7/s1, Figure S1: Medline search strategy, Figure S2: Risk of bias summary tables.
