# Supplementary material for: A Systematic Review of Organic Versus Conventional Food Consumption: Is There a Measurable Benefit on Human Health?
Source: Nutrients. 2019 Dec 18;12(1):7. doi: 10.3390/nu12010007 (PMC7019963; doi:10.3390/nu12010007)
Supplement: Supplementary file 1 [file nutrients-12-00007-s001.pdf]

Supplementary Figure S1: Medline Search Strategy

Search strategy

MEDLINE strategy (run - 30/1/19)

1. MH organic agriculture or food, organic or pesticide residues
2. AB organic* or bio-dynamic* or biodynamic* or bio-organic* or bioorganic* or bioorganic* or ecologically or naturally or biologically
3. TI organic* or bio-dynamic* or biodynamic* or bio-organic* or bioorganic* or bioorganic* or ecologically or naturally or biologically
4. S1 OR S2 OR S3
5. AB conventional* or nonorganic* or non-organic*
6. TI conventional* or nonorganic* or non-organic*
7. SU conventional* or nonorganic* or non-organic*
8. S5 OR S6 OR S7
9. S4 AND S8
10. MH fruit or vegetables or meat or dairy products or eggs or whole grains or bread or crops, agricultural or meals or nuts or seeds or raw foods or flavonoids or polyphenols or carotenoids
11. AB fruit or vegetables or meat or dairy or eggs or “whole grain*” or wholegrain* or beef or produce or meals or diet or food or cereal* or cruciform or grain or seed or seeds or nuts or nut or honey or crop or crops or granary or wholewheat or legume* or herbs or spices or juice* or beverage* or dairies or poultry or pork or milk or flavonoid* or polyphenol* or carotenoid*
12. TI fruit or vegetables or meat or dairy or eggs or “whole grain*” or wholegrain* or beef or produce or meals or diet or food or cereal* or cruciform or grain or seed or seeds or nuts or nut or honey or crop or crops or granary or wholewheat or legume* or herbs or spices or juice* or beverage* or dairies or poultry or pork or milk or flavonoids or polyphenols or carotenoids
13. S10 OR S11 OR S12
14. AB produce* or raise or raising or grown* or cultivate* or raised or farm* or production
15. TI produce* or raise or raising or grown* or cultivate* or raised or farm* or production
16. SU produce* or raise or raising or grown* or cultivate* or raised or farm* or production
17. S14 OR S15 OR S16
18. S9 AND S13 AND S17
19. "health food"
20. PT comparative study
21. S9 OR S18 OR S19
22. S20 AND S21
23. S9 AND S13
24. S9 AND S17
25. S22 OR S23 OR S24
26. MH respiratory tract diseases OR arthritis OR asthma or eczema or reproductive health or lactation or breast feeding or semen or cardiovascular disease or osteoporosis or neoplasms or chronic disease or health or disease
27. AB respiratory or allerg* or asthma or eczema or lactat* or "breast milk" or "reproductive health" or semen or fertility or "quality of life" or arthritis or "irritable bowel" or "nutritional status" or "breast feeding" or breastfeeding or "weight loss" or "weight gain" or "body composition" or diabetes or cardiovascular or osteoporosis or "bone density" or neoplasm* or cancer or health or disease or autoimmune or urine or “urinary excretion” or blood
28. TI respiratory or allerg* or asthma or eczema or lactat* or "breast milk" or "reproductive health" or semen or fertility or "quality of life" or arthritis or "irritable bowel" or "nutritional status" or "breast feeding" or breastfeeding or "weight loss" or "weight gain" or "body composition" or diabetes or cardiovascular or osteoporosis or "bone density" or neoplasm* or cancer or health or disease or autoimmune or urine or “urinary excretion” or blood
29. S26 OR S27 OR S28
30. S25 AND S29

Results = 1634

Key: AB abstract; MH MeSH heading; PT publication type; SU subject; TI title

Supplementary Figure S2: Risk of bias assessments

1. Clinical trial ROB - Cochrane Collaboration's tool for assessing risk of bias in randomised trials [1]

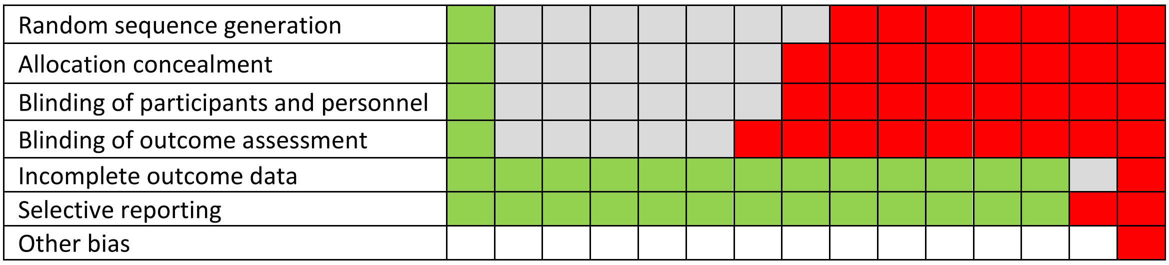

Key: green = low risk of bias; grey = unclear risk of bias; red = high risk of bias

	Random sequence generation	Allocation concealment	Blinding of participants and personnel	Blinding of outcome assessment	Incomplete outcome data	Selective reporting	Other bias
Caris-Veyrant (2004)	?	?	?	-	+	+	
Stracke (2009)	?	?	?	?	+	+	
Stracke (2010)	?	?	?	?	+	+	
Briviba (2007)	?	?	?	?	+	+	
Grinder-Pedersen (2003)	?	?	?	?	+	+	
Ackay (2004)	-	-	-	-	-	-	
Lu (2006)	-	-	-	-	+	+	
Lu (2008)	-	-	-	-	+	+	
Di Renzo (2007)	-	-	-	-	?	-	
De Lorenzo (2010)	-	-	-	-	+	+	
Soltoft (2011)	+	+	+	?	+	+	
Tolado (2016)	?	-	-	-	+	+	
Goen (2017)	-	-	-	-	+	+	-
Bradman (2015)	-	-	-	+	+	+	
Oates (2014)	?	?	?	-	+	+	

2. Cohort studies ROB – Newcastle Ottawa Quality Assessment Form for Cohort Studies [2]

	Brantsaeter 2016	Torjusen 2016	Christensen 2013	Kummeling 2008	Kesse-Guyot 2017	Stenius 2011	Buscail 2015	Bradbury 2015	Baudry 2018 (x3)	Chiu 2018
Selection										
Representative of the exposed cohort	X	X	X	X	X	X	X	X	X	X
Selection of the non-exposed cohort	X	X	X		X	X	X	X	X	X
Ascertainment of exposure	X	X								
Outcome of interest not present at beginning	X	X		X		X	X	X		X
Comparability										
Comparability of cohorts	X	X	X	X	XX	X	X	X	XX	X
Outcome										
Assessment of outcome	X	X	X	X		X		X	X	X
Adequate follow-up length	X	X	X	X	X	X	X	X	X	X
Adequate subjects retained to follow-up	X	X	X	X		X	X	X	X	X
**Rating**	**8**	**8**	**6**	**6**	**5**	**7**	**6**	**7**	**7**	**7**

Thresholds for converting the Newcastle-Ottawa scales to AHRQ standards (good, fair, and poor):

Good quality: 3 or 4 stars in selection domain AND 1 or 2 stars in comparability domain AND 2 or 3 stars in outcome/exposure domain

Fair quality: 2 stars in selection domain AND 1 or 2 stars in comparability domain AND 2 or 3 stars in outcome/exposure domain

Poor quality: 0 or 1 star in selection domain OR 0 stars in comparability domain OR 0 or 1 stars in outcome/exposure domain

3. Cross-section ROB - Specialist Unit for Review Evidence (SURE) for Cross-sectional Studies [3]

	Rist 2007	Mueller 2010	Juhler 1999	McGuire 2016	Curl 2003	Curl 2015	Baudry 2017	Jensen 1996
Design clearly stated	✓	✓	✓	✓	✓	✓	✓	✓
Clear study question	✓	✓	✓	✓	✓	✓	✓	✓
Setting, location, dates provided	✓	✓	✓	✓	✓	✓	✓	X
Participants fairly selected	✓	✓	✓	✓	X	✓	✓	X
Participant characteristics provided	✓	✓	✓	✓	✓	✓	✓	X
Appropriate measures used	✓	✓	✓	✓	✓	✓	✓	✓
Description of study size	✓	X	✓	✓	✓	✓	✓	✓
Statistical methods well described	✓	✓	✓	✓	✓	✓	✓	X
Participant eligibility information provided	✓	✓	✓	✓	✓	✓	✓	X
Results well described	✓	✓	✓	✓	✓	✓	✓	X
Sponsorship/conflict of interest detailed	✓	✓	✓	✓	✓	✓	✓	✓
Limitations identified	✓	X	✓	✓	✓	✓	✓	✓

References

1. Higgins, J.P.T.; Altman, D.G.; Gøtzsche, P.C.; Jüni, P.; Moher, D.; Oxman, A.D.; Savović, J.; Schulz, K.F.; Weeks, L.; Sterne, J.A.C. The Cochrane Collaboration's tool for assessing risk of bias in randomised trials. *BMJ* **2011**, *343*.
2. Wells, G.; Shea, B.; O'Connell, D.; Peterson, J.; Welch, V.; Losos, M.; Tugwell, P. The Newcastle-Ottawa Scale (NOS) for assessing the quality of nonrandomised studies in meta-analyses. Availabe online: http://www.ohri.ca/programs/clinical_epidemiology/oxford.asp (accessed on 22nd March 2019).
3. Specialist Unit for Review Evidence (SURE). Questions to assist with the critical appraisal of cross-sectional studies. Availabe online: https://www.cardiff.ac.uk/specialist-unit-for-review-evidence/resources/critical-appraisal-checklists (accessed on 23rd Jan 2019).
